# Experimental Researches on the Properties and Functions of the Nervous System in Vertebrated Animals

**Published:** 1843-10

**Authors:** 


### ART. VII.

*Recherches Expérimentales sur les Propriétés et les Fonctions du Système Nerveux dans les Animaux Vertébrés.* Par P. FLOURENS, Membre de l'Académie Française, Secrétaire perpétuel de l'Académie Royale des Sciences, etc. etc. Seconde Edition, corrigée, augmentée, et entièrement refondue.—*Paris*, 1842. 8vo, pp. 516.

*Experimental Researches on the Properties and Functions of the Nervous System in Vertebrated Animals.* By P. FLOURENS, Member of the French Academy, Perpetual Secretary of the Royal Academy of Sciences, &c. &c. Second Edition, corrected, enlarged, and entirely recast.—*Paris*, 1842.

THE work of M. Flourens, of which this new edition has recently appeared, is well known to professed physiologists as one of the standards of their science. It appeared about the same time that the discoveries of Sir C. Bell were first made known to the world; and the reputation which it might otherwise have gained was, in this country at least, obscured by the more brilliant rays of our own luminary; but on the continent it has been more fully appreciated, and we believe that to it is mainly due the high position which M. Flourens has obtained in the scientific circles of Paris. In Britain his discoveries have been gradually incorporated with the "received text" of physiological science, without due attention to their source; and we think it but a deserved tribute to his merits to call the attention of our readers to the acknowledged merits of his former researches, as well as to notice those of more recent date and greater novelty.

The volume before us not only includes the whole of the work which appeared under the same title in 1824, together with the supplementary papers which were published in the following year, and others which have been since read before the academy, and which have been published in their transactions; but also the results of some additional researches of great interest which have been more recently made by M. Flourens. To these last we shall presently draw the more particular attention of our readers; but we shall commence by giving a general account of the results of his former inquiries, which the resumé drawn up by himself will enable us to do in his own words. It is necessary that we should explain that M. Flourens has slightly changed his phraseology since the first publication of these memoirs. The discoveries of Sir C. Bell have led him (as we anticipated, see vol. V. p. 537,) to separate more completely the properties of the *nerves* from those of the *spinal cord.* For the first he retains the term *excitability,* whilst to the latter he gives the appellation of *sensibility;* but he expressly states that by this term he means simply the power of "receiving and transmitting impressions," which is precisely Dr. M. Hall's "Reflex Function." Indeed we find a similar use of the word among the French writers in general. Thus, in one now before us,* we meet with the following passage: "All living organized beings are *sensible* or *irritable*, having the power of acting of themselves, and of reacting on the causes which tend to modify their existence. But all are not *sentient*, that is to say, they are not *conscious* of that

* Analyse Physiologique de l'Entendement Humain. Par J. C. Collineau.

which takes place around and within them." The term *perception*, to which we are accustomed to attach a higher notion, is the one by which French physiologists usually designate what *we* understand by *sensation*, or the *consciousness of an impression.* The following is M. Flourens's own general estimate of the tendency of his inquiries. It is to be remembered that they were *contemporaneous* with those of Sir C. Bell, and that in the following summary, therefore, these are only alluded to where they bear upon the author's own :

"It was early perceived that the nervous system is at the same time the organ by which the animal receives *sensations* (impressions), the organ by which it executes or determines its *movements*, the organ by which it *perceives* and *wills*. But does the faculty of *willing* and *perceiving* reside in the same parts with the property of *feeling*? Does the property of *feeling* (receiving impressions) reside in the same parts with the property of movement? Are thinking, feeling, moving, but one property, or are they three distinct properties, having separate organs for their manifestation? These important questions, debated during so many ages, still remained for solution.

"My experiments showed, in a most decisive manner, that there are three essentially distinct properties in the nervous system; that of *perceiving* and *willing*, that of *feeling* (receiving impressions), and that of *moving;* that these three properties have seats as distinct as their effects; and that their organs are separated from each other by definite limits.

"The nerves, the spinal cord, the medulla oblongata, and the tubercula quadrigemina or bigemina, are the only immediate *excitors* of muscular contraction; the cerebral lobes are restricted to the power of *willing* this movement, and do not immediately excite them; and further, that in the spinal cord and in the nerves, the parts which *excite* movements are not the same as those which are *sensible*, and those which are sensible have not the power of exciting movement. (Sir C. Bell.)

"There are, then, in the nervous system three properties essentially distinct; one, that of *perceiving* and *willing;* in other words, *intelligence ;* another, that of *receiving* and *transmitting impressions*, or *sensibility;* the third, that of *immediately exciting muscular contraction*, which I propose to call *excitability*. Further, there resides in the cerebellum a property, of which there had been previously no idea in physiology; and which consists in the *co-ordination* of the movements *willed* by certain parts of the nervous system, and *excited* by others. The nerve directly excites the contraction of the muscle; the spinal cord unites the separate contractions into regular movements; and the cerebellum co-ordinates these movements into the regular movements of locomotion,—walking, running, flying, &c.; whilst by the cerebral lobes, the animal *perceives* (feels) and wills." (Preface, pp. x-xiii.)

By this general statement of the opinions of M. Flourens, and the explanation of his phraseology, our readers will be prepared to follow us through his chapter on the *Laws of nervous action ;* which, being itself a condensed summary of the results of his experiments, we shall translate without abridgment :

"Three grand laws govern the action of the nervous system: the first is the *speciality* (distinctness) of action; the second is the *subordination of the nervous functions ;* the third is the *unity of the nervous system.*

"*Speciality of nervous action.* I. It has been shown that every essentially distinct part of the nervous system has a function or mode of acting, which is equally distinct. The cerebrum does not act in the manner of the cerebellum, nor the cerebellum like the medulla oblongata, nor the medulla oblongata like the spinal cord and nerves. II. Each part of the nervous system has, then, a peculiar and special action; that is, a different action from the others; and we have further seen in what this speciality consists. In the cerebral lobes resides the faculty by which the animal thinks, wills, recollects, judges, becomes conscious of sensations, and commands its movements. From the cerebellum is derived the faculty which co-ordinates the movements of locomotion; from the tubercula bigemina or quadrigemina, the primordial principle of the action of the optic nerve and retina; from the medulla oblongata, the motor or exciting principle of the respiratory movements; and, lastly, from the spinal cord itself, the faculty of blending or associating into combined movements the partial contractions immediately excited by the nerves in the muscles. III. The important fact of the speciality of action of the different parts of the nervous system—a fact, towards the demonstration of which the noblest efforts of physiologists were for a long time directed, is then henceforth established by direct observation, and the result demonstrated by experience.

*Speciality of the nervous properties.* I. There are three properties of the nervous system essentially distinct; 1, that of exciting muscular contraction, or *excitability;* 2, that of receiving and transmitting impressions, or *sensibility;* 3, that of perceiving and willing, or *intelligence.* II. And each of these properties has a distinct seat in a determinate organ. *Excitability* resides in the anterior column of the spinal cord, and in the nervous fibres which arise from it. *Sensibility* has its seat in the posterior columns of the spinal cord, and in the nerves that originate (or terminate) in it. *Intelligence* is restricted to the cerebral hemispheres.

*Office of each part of the nervous system in locomotion.* I. No movement is immediately derived from the will. The will is only the provoking (exciting) cause of certain movements; and is never the efficient cause of any. When an animal wills to move its arms, its leg, or any other part, it immediately moves it; but it is *not* the will which animates the muscles of the part moved, excites them to action, and combines their movements; and these functions are not performed by the cerebral hemispheres in which the will resides. II. The direct cause of muscular contractions resides particularly in the spinal cord and its nerves; the cause which co-ordinates the movement of different parts, resides exclusively in the cerebellum. III. Here then, are three distinct phenomena in a voluntary movement: 1, the willing of the movement, which resides in the cerebral lobes; 2, the co-ordination of the various parts that concur to produce this movement, which resides in the cerebral hemispheres; 3, the excitation of the muscular contractions, which has its seat in the spinal cord and its nerves. IV. Since these three principal phenomena are essentially distinct, and have their seat in three organs which are also essentially distinct, we see the possibility of abolishing one of them, the will for example, without destroying the others; or of abolishing at once the will and the co-ordination, without interfering with the contraction. V. Of this, the experiments contained in the present work afford complete evidence. An animal deprived of its cerebral lobes no longer moves spontaneously or voluntarily; but it moves with co-ordination, and quite as regularly as when these lobes are present. An animal deprived of its cerebellum, on the contrary, loses all power of co-ordinating its movements. Nevertheless all the parts of such an animal, its head, trunk, and extremities, perform movements of their own; but their motions are no longer co-ordinated, nor is a combined result obtained. Such an animal no longer walks, no longer flies, nor holds itself upright: not that it has lost the use of its legs and wings, but because the co-ordinating power no longer exists. In a word, all the partial movements still exist; but the co-ordination of these movements is lost. VI. What I have said of the cerebellum, in reference to the co-ordinate movements of locomotion, may be said of the medulla oblongata, with reference to the co-ordinate movements of conservation. Whilst this part of the medulla exists, they remain; when it is destroyed, they cease. [Under this head, M. Flourens points to the movements of respiration, deglutition, &c.] Hence their regulating principle, or *primum mobile,* has its residence there. VII. With respect to the spinal cord, its function is restricted to the union of muscular contractions, the first elements of all movements, into combined motions; and although all the nerves which determine these contractions and movements issue from it, it is nevertheless not in *it* that the wonderful faculty resides by which these contractions and movements are co-ordinated into determinate actions, such as leaping, flying, walking, running, standing, &c., or inspiration, crying, yawning, &c. This faculty resides in the cerebellum as to the former of these movements, and in the medulla oblongata as to the latter. VIII. The movements of respiration, crying, yawning, &c. are commonly termed *involuntary*, in opposition to the movements of locomotion, which are termed *voluntary.* We have just seen in what sense the term *voluntary*, as applied to certain movements, must be understood. The will is never anything else than the exciting, external, occasional cause of these movements; still it can provoke them, regulate their energy, and determine their object. Thus an animal can, at will, either move or remain still, can walk quickly or slowly, and in whatever direction it please. It is then absolute master, not of the mechanism of its movement, but of the movement itself. It is the same as to running and leaping, which are only a more rapid kind of walking; as to flying, swimming, crawling, which are only different species of progression; as to standing, which is but a part of walking; and, in a word, as to all the movements of locomotion or translation. On the other hand, respiration, crying, yawning, certain dejections, &c. only depend upon the will up to a certain point, and in certain cases. In general, all these movements take place without its either perceiving them or participating in them; and often in complete opposition to it. Lastly, the movements of the heart and intestines are absolutely and totally removed from the influence of the will. With regard to the will, therefore, as with respect to the mechanism and to the organs of movement, there are three kinds of movement essentially distinct from each other. Those of the first class are entirely under its control; those of the second are so only in part; and those of the third are not so at all.

"*Subordination of the functions of the nervous system.* I. These functions are to a certain extent under subordination to each other. II. There are, in the nervous system, some parts which act spontaneously or of themselves; and there are others which act only in subordination to the rest, or when impelled by them. III. The subordinate parts are the spinal cord and nerves; the *regulating* and *primordial* portions are the medulla oblongata, the seat of the principle which determines the movements of respiration; the cerebellum, the seat of the principle which co-ordinates the movements of locomotion; and the cerebral lobes, the exclusive seat of intelligence.

"*Unity of the nervous system.* I. Not only are all the parts of the nervous system thus mutually connected, but they are all subordinated to one organ. II. The nerves and the spinal cord are all subordinated to the encephalon; the nerves, the spinal cord, and the encephalon are subordinated to the medulla oblongata, or more precisely to the vital and central point of the nervous system, placed in the medulla oblongata. III. It is with this point, placed in the medulla oblongata, that all the other parts of the nervous system must be connected that their functions may be *exercised.* The principle of the *exercise* of nervous action passes along the nerves to the spinal cord, and from the spinal cord to this point; and, having passed this point, it moves in a retrograde direction from the anterior parts of the encephalon to the posterior, and from the posterior to this point again.

"*Unity of the cerebrum, or of the organ which is the seat of intelligence.* I. The unity of the cerebrum, or of the organ which is the seat of intelligence, is one of the most important results of this work. II. The organ, the seat of intelligence, is one. III. In fact, not only do all the perceptions, all the volitions, all the intellectual faculties, reside exclusively in this organ; but all these faculties occupy the same place in it. As soon as one of them disappears by lesion of a given part of the cerebrum, they all disappear. As soon as one of them returns by the cure of the injury, all return. The faculty of perceiving and willing, constitutes, therefore, a faculty which is essentially *one;* and this single faculty resides in a single organ." (pp. 235-44.)

We are inclined to think that, in these latter propositions, M. Flourens attaches too much importance to the medulla oblongata as a distinct and peculiar organ; but it must be confessed that there is much that yet remains to be explained in regard to the action of the brain upon the motor nerves. It may even, indeed, be questioned whether any of these nerves proceed directly from the cerebrum; and whether they have not all their real termination in the spinal cord. Upon this view, the same fibres might be excited to action either by a stimulus applied to the spinal cord itself, or by one conducted to it by an afferent nerve, or by one produced by the influence of the will through the brain ; just as muscular fibre may be excited to perform the same contraction by various stimuli applied in different ways. This we believe to be the idea of Müller, though we must confess ourselves at a loss to form a definite notion of his opinions, owing to a great want of precision in the expression of them. But however it is to be explained, the *fact* that irritation of the cerebral ganglia does not in any case produce muscular movements, except when the upper part of the medulla oblongata is affected, remains as a proof that some *break* intervenes between the cerebrum and the muscle which is to be called into action ; and numerous phenomena of *semi-voluntary* motion,—by which term we may designate those actions which were at first purely voluntary, but have since become more or less independent of the will through being rendered habitual by frequent repetition, such as the playing on a musical instrument,—lead to a similar conclusion.

That the cerebral ganglia are the instruments of perception, memory, the intellectual faculties, and the will, may be regarded as a position fixed by the researches of M. Flourens. That the motor principle is *immediately* derived from the spinal cord and medulla oblongata, seems also to be a legitimate deduction from his experiments; and it was by him that the principle was first brought prominently forwards, that this motor principle may be excited to action, by an external impression that does not produce sensation. The determination of the functions of the cerebellum had been previously made, without the knowledge of M. Flourens, by Rolando ; and the near coincidence of the two series of results is sufficient to show that, even if neither precisely expresses the truth, neither is far from it. Similar experiments have been subsequently made by Hertwig; with corresponding results. All these experiments lead to the conclusion, that the cerebellum is the organ by which the simple motions of the several parts of the body are blended together, for the performance of the actions of locomotion, and for the balancing of the body when at rest; and this conclusion harmonises so well with the evidence supplied by comparative anatomy, as to the relative development of the cerebellum in groups of animals that are distinguished by differences in the number and variety of such combined actions, that we cannot but regard it as entitled to take rank as a physiological truth. Our phrenological readers need not imagine that the admission of this view necessarily militates against the doctrine of Gall, respecting the seat of the sexual impulse ; since it is quite possible that the two functions may coexist in the cerebellum ; and there are phrenologists of eminence, who have found themselves compelled by the weight of evidence to admit the truth of Flourens' views. It is not by any means a satisfactory mode of getting rid of such evidence, to say that no value can be attached to vivisections ; since an animal that has undergone so severe a mutilation of its nervous centres cannot be expected to keep its balance, walk, fly, &c. For is not the removal of the cerebral hemispheres an operation of much greater severity? And yet after this, animals may live for months, standing, walking, flying, &c., but doing all as if in a perpetual sleep. M. Flourens relates that a cock from which nearly half the cerebellum had been removed, frequently attempted to have intercourse with hens in whose company he was left; showing that the sexual appetite was not destroyed by this severe mutilation. But he could never succeed, for want of power to execute the necessary combined movements, and to retain his equilibrium.

In the subsequent part of the volume we find several papers on detached subjects of much interest; but which, having been long before the public, do not now require particular notice. One of these contains the results of a series of experiments on the reunion of nerves; in which satisfactory evidence is given of the gradual restoration of function, even when large trunks have been divided; and of renewal of the nervous substance, even when a considerable portion of it has been removed. There is also an interesting paper on the movement of the brain, observed when a portion of it is laid bare in a living animal. This is shown to depend entirely upon the distension of its vessels with blood; and hence to be in part coincident with the arterial pulse, and in part with the respiratory or venous pulse.

The most remarkable novelty in the volume is that which relates to the functions of the semi-circular canals of the internal ear, and of the nerve by which they are supplied. This nerve is regarded by M. Flourens to have an office quite distinct from that of ministering to audition; and to be concerned in regulating the locomotive actions of the body. His former volume contained a memoir on the "fundamental conditions of audition," which was presented to the Royal Academy at the end of 1824. In this memoir, M. Flourens attempts to prove that the cochlea is the essential part of the internal ear; the sense of hearing not being destroyed so long as this remains entire, even though the vestibule and the semi-circular canals have been laid open and their nervous expansion completely destroyed. The laying open of the interior of the vestibule does not, according to him, perceptibly impair the acuteness of the sense, and the rupture of the semi-circular canals seems only to render hearing painful, without making it less acute, whilst it also causes very remarkable movements of the head.

Now we must remark upon the conclusions arrived at in this memoir, that they do not appear to us by any means satisfactory. The evidence of the comparative effects produced by the various operations upon the sense of hearing, is extremely imperfect, and does not appear to us to warrant the deductions which M. Flourens has drawn from it. Comparative anatomy affords much more satisfactory indications on the subject of the essential part of the organ of hearing; for, in descending the animal scale, we find one accessory part removed after another, until there remains the *vestibule* only; and this is evidently, therefore, the chief seat of the sense.

But the peculiar function of the semi-circular canals can only be determined by experiments, and those of M. Flourens are worthy of attentive study. He submitted to the commissioners of the academy, at the commencement of the year 1825, a pigeon, in which he had cut the horizontal semi-circular canal on the two sides, about two months previously. The immediate effect of the operation had been to cause a rapid jerking motion of the head from side to side, which continued with very little interruption; and also a tendency to turn to one side, which manifested itself whenever the animal attempted to walk forwards. The horizontal movement of the head, though less sudden and violent than it was immediately after the operation, still continued, and manifested itself particularly when the animal was excited to motion. The revolving action also was renewed whenever the animal was caused to move with rapidity. This revolution took place sometimes to the left, and sometimes to the right; but most frequently to the right. The movements continued with the same intensity for several months, during which period the animal retained its faculties, and being fed with care, it became very fat. Its head was then examined, and it was found that the two canals which had been cut, were obliterated at the points of section, and that no part of the train appeared to have suffered any lesion. M. Flourens has since repeated these experiments in a great variety of ways, and the results are contained in two memoirs, one, on the semi-circular canals in birds; and the other, on those of mammalia; which were read before the academy in 1828. The following is a summary of them:—Section of the horizontal canal on the two sides, in pigeons and other birds, is followed by a violent horizontal movement of the head. Section of a vertical canal, whether the inferior or superior, on both sides, is followed by a violent vertical movement of the head. And section of the horizontal and vertical canals at the same time, causes horizontal and vertical movements. Section of either canal on one side only, is followed by the same effect as when the canal is divided on both sides; but this is much inferior in intensity. In rabbits, section of the horizontal canal is followed by the same movement as in pigeons, and it is even more constant though less violent. Section of the anterior vertical canal causes the animal to make continual forward *somersets;* whilst section of the posterior vertical canal occasions continual backward somersets. The movements cease when the animal is in repose; and they recommence when it begins to move, increasing in violence as its movement is more rapid. The connexion of the particular movements, with section of the particular canals, is shown by the constancy with which the same effects follow the same lesion, in different animals.

These facts are probably familiar to many of our readers; and we merely recall them now, that the reasoning founded on them by M. Flourens in the two chapters on "the direction of the movements of the animal, governed by the direction of the fibres of the encephalon," and on the "moderating forces of the movements," which are here published for the first time, may be intelligible. These experiments were reported on to the academy by M. Cuvier, who pointed out the analogy between their results, and those which had been obtained by the experiments of Magendie, on section of the pons varolii, as well as those which the experiments of M. Flourens on the cerebellum had afforded. Injury of the cerebellum on one side, section of its crus on one side, and section of the horizontal semi-circular canal, all produce the same result; and it was natural, therefore, to infer that there is some condition common to all by which this result is brought about. This idea has been followed up by M. Flourens, who points out that each of the crura cerebelli consists of three fasciculi of fibres, which correspond to the three directions of movement:—1, The transverse fibres which embrace the medulla oblongata, forming the pons varolii;—2, The postero-anterior peduncles, which pass from the cerebellum towards the tubercula quadrigemina, forming the processus a cerebello ad testes, and becoming continuous with the crura cerebri;—3, The antero-posterior fibres, which pass backwards to the medulla spinalis, forming the corpora restiformia. Now it has been shown by experiment that if the pons varolii, containing the transverse or lateral fibres of the cerebellum, be divided, the animal rolls over sideways upon itself. If the crura cerebri, containing the anterior fibres, be cut, the animal forcibly precipitates itself forward, in the same manner as if the anterior vertical semi-circular canal were divided. And if the posterior peduncles of the cerebellum be divided, the animal tends to make a series of backward somersets, in the same manner as if its posterior vertical semi-circular canal were divided. It has been ascertained by M. Flourens that lesions of the portions of the cerebellum, from which these peduncles respectively proceed, produce the same effects. The relation to which this comparison points is certainly a very curious one.

The acoustic or auditory nerve, according to our author, is not a simple nerve, but a complex one, consisting of two essentially distinct trunks, the nerve of the cochlea, and the nerve of the semi-circular canal. The first of these is the true auditory nerve, the cochlea being (according to M. Flourens) the true seat of the sense of hearing. He assures us that he has on several occasions succeeded in destroying the cochlea of rabbits without injuring the vestibule; and that the sense of hearing was totally destroyed thereby. We must take the liberty of regarding these experiments as very unsatisfactory, since their results may be influenced by a great variety of causes; and of preferring the "experiments prepared for us by nature," who takes away the cochlea in amphibia and fishes, so that whatever power of audition these animals possess must be due to the vestibule and semi-circular canals. Still lower down in the scale the semi-circular canals disappear, and the vestibule alone remains. This is the case in some of the lowest fishes, and in the invertebrata. Let it be observed, however, that our argument does not in any way go against the peculiar function ascribed to the semi-circular canals by M. Flourens; but only to show that he has by no means established their absence of participation in the sense of hearing.

The nerve of the semi-circular canals consists of three fasciculi, each of which has a distinct origin and a distinct termination; whilst the true auditory nerve has but a single origin. One of these roots is derived from the transverse peduncles of the cerebellum; another from the anterior peduncles which enter the crura cerebri; and the third from the posterior peduncles, or corpora restiformia. These three fasciculi are distributed to the horizontal, anterior vertical, and posterior vertical canals respectively. A full account of their origin and course is promised by M. Flourens in a forthcoming work on the Anatomy of the Brain. Hence it is evidently from the nervous connexions with these parts of the encephalon, that the semi-circular canals derive the remarkable power which they have been shown to possess.

M. Flourens then goes on to inquire into the mode in which these curious movements are occasioned. He considers that it cannot be said, with justice, that section of such and such a canal, or of such and such a fasciculus of fibres, *determines* the effect, but that it rather allows it to manifest itself. For it is the *absence* of the horizontal semi-circular canal which produces the lateral movement, and its integrity seems to restrain that movement.

"In fact, the action of the semi-circular canals, and of the fibres of the encephalon which are opposed to each other in direction, is rather an action which moderates, governs, controls, than a force which urges or determines. This force is composed of several. There are in the semi-circular canals, and in the opposed fibres of the encephalon, several forces which control and moderate; in fact, there are as many *moderating* forces as there are opposed directions of movement. Thus the anterior vertical canal, and the anterior peduncles of the cerebellum, moderate the forward movement; the posterior vertical canal, and the posterior peduncles of the cerebellum, moderate the backward movement; whilst the horizontal canal, and the lateral fibres of the cerebellum, moderate the lateral movement." (pp. 497-8.)

The nervous system, then, according to M. Flourens, is not only the *exciting principle* of the movements of the body, but is also the *regulating* and the *moderating* principle. These principles have their seat in distinct parts of the system. The *exciting* effect is produced by all those parts, which, by being pricked or irritated, immediately cause muscular contractions; that is, by the spinal cord, the medulla oblongata, and by the nerves. The *regulating* effect emanates from the cerebellum. The *moderating* effect resides in the semi-circular canals, and in the *opposing fibres* of the encephalon.

Now we are very far from being satisfied that any such new principles as those introduced by M. Flourens are required to explain what are certainly the very curious effects to which he has directed attention. It seems to us quite possible that the nerve of the semi-circular canals may be connected with the government of the motions of the body, without being the less a nerve of sense; just in the same manner as the optic nerve is connected with the *consensual* movements of the eyes, and with the contraction and dilatation of the pupil. There is undoubtedly much to be known respecting these consensual movements, which seem to be distinct from the reflex, in that they necessarily involve *sensation;* and which seem to bear a closer resemblance to those of the emotional and instinctive group. Their study, if prosecuted in a right direction, will amply repay the physiological inquirer; and he cannot do better than commence by repeating the experiments of M. Flourens, with all the precautions indicated by him, and others that will suggest themselves. We must own that we have not sufficient confidence in experiments of this kind to take upon trust the results which have been obtained by a single inquirer; for the recent history of neurological research affords abundant reason for hesitation in the reception of such results, until they have been confirmed by further inquiries, instituted by competent and unprejudiced experimenters.

